# Supplementary figures and images for: Evaluation of putative reference genes for quantitative real-time PCR normalization in Lilium regale during development and under stress
Source: PeerJ. 2016 Mar 21;4:e1837. doi: 10.7717/peerj.1837 (PMC4806604; doi:10.7717/peerj.1837)

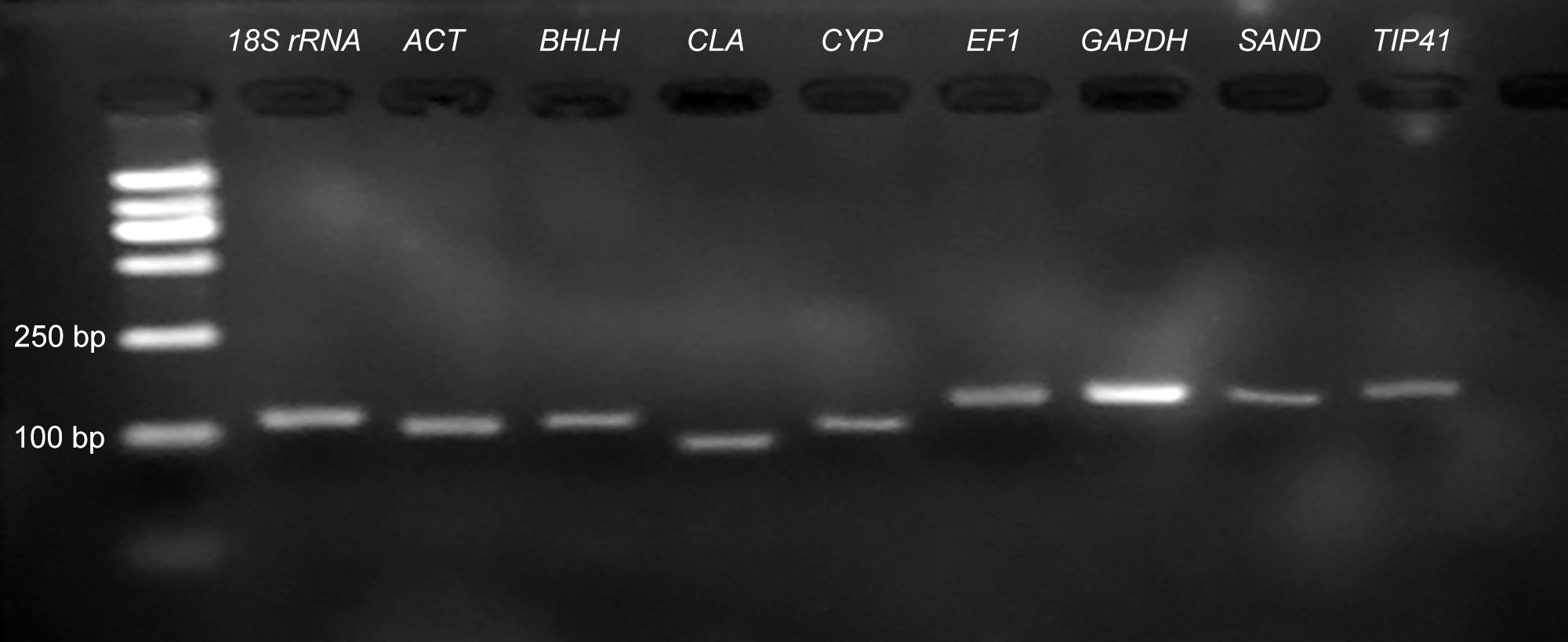

Supplement: Supplemental Information 1 [file peerj-04-1837-s001.png]

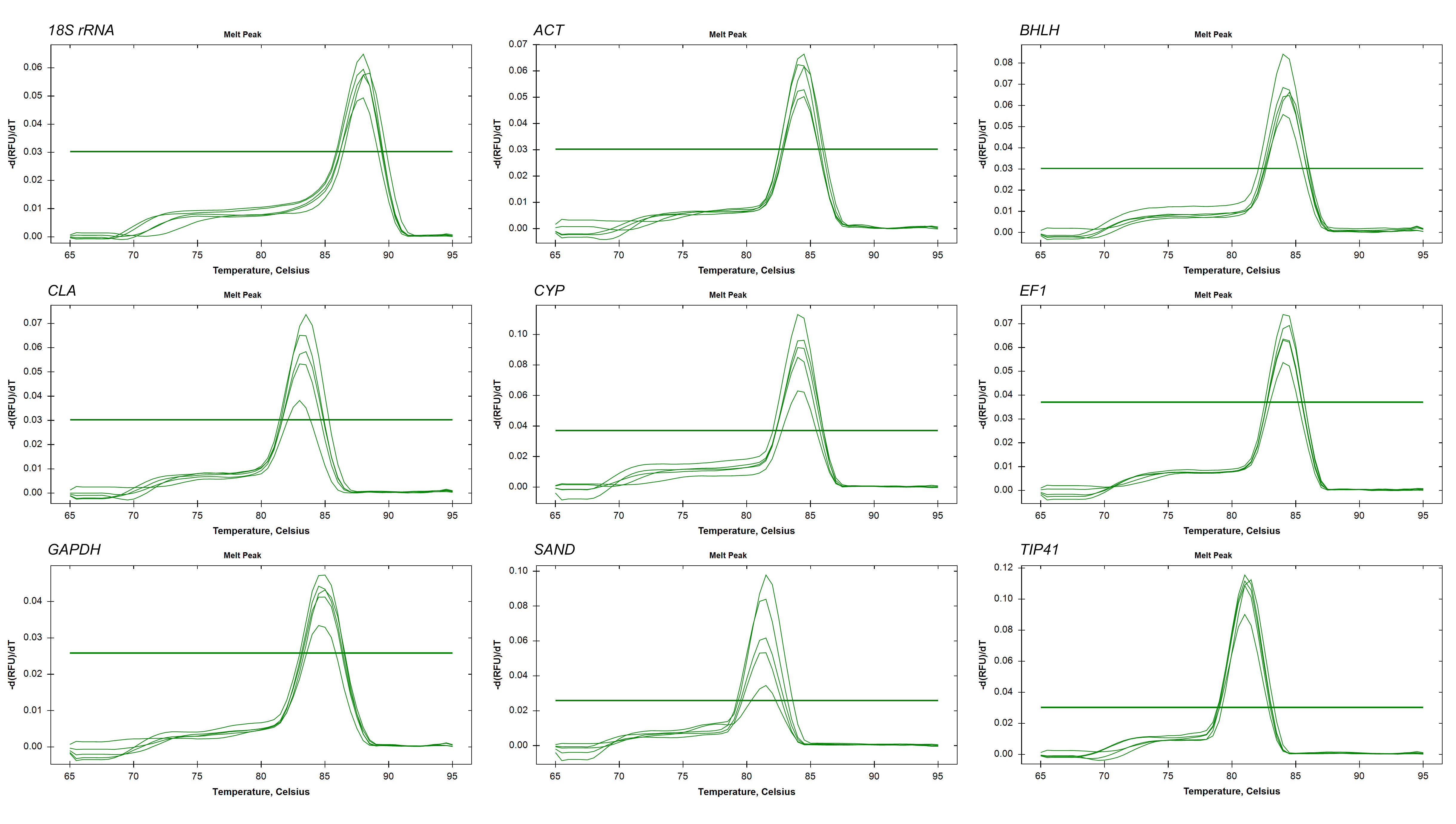

Supplement: Supplemental Information 2 [file peerj-04-1837-s002.png]
