# Supplementary material for: Evaluation of putative reference genes for quantitative real-time PCR normalization in Lilium regale during development and under stress
Source: PeerJ. 2016 Mar 21;4:e1837. doi: 10.7717/peerj.1837 (PMC4806604; doi:10.7717/peerj.1837)
Supplement: Supplemental Information 3 — hpi, hours post inoculation. [file peerj-04-1837-s003.docx]

**Table S1.** RPKM values for the nine selected genes in RNA-Seq data of *Lilium regale* after inoculation with *B. elliptica*

| Gene | 0 hpi | 4 hpi | 12 hpi | 24 hpi |
| --- | --- | --- | --- | --- |
| *18S rRNA* | 49.66 | 59.66 | 47.84 | 77.59 |
| *ACT* | 275.44 | 268.60 | 271.28 | 232.39 |
| *BHLH* | 2.91 | 3.74 | 1.97 | 3.04 |
| *CLA* | 26.40 | 27.96 | 27.74 | 26.73 |
| *CYP* | 6.00 | 6.72 | 5.47 | 7.07 |
| *EF1* | 125.38 | 123.29 | 112.25 | 111.69 |
| *GAPDH* | 508.02 | 675.50 | 795.91 | 635.02 |
| *SAND* | 91.61 | 98.70 | 98.19 | 108.14 |
| *TIP41* | 17.52 | 17.97 | 15.37 | 16.94 |

hpi: hours post inoculation.
