# Supplementary material for: Evaluation of putative reference genes for quantitative real-time PCR normalization in Lilium regale during development and under stress
Source: PeerJ. 2016 Mar 21;4:e1837. doi: 10.7717/peerj.1837 (PMC4806604; doi:10.7717/peerj.1837)
Supplement: Supplemental Information 4 [file peerj-04-1837-s004.docx]

> *18s RNA*

GCAGAATCCCGTGAACCATAGGAGCTTGGAACTGATAAAGCAAAGCAAGCTGCATTGCTTGGTGGAGGTGGCAGCAAAGCACAGAATGAACGAGCTGCCGCACTAGCTGCGATTTTAGCTGCAAGATGGATGCGGAGATTGGC

> *ACT*

CCCATTGAGCACGGCATTGTCAGCAACTGGGATGACATGGAGAAGATCTGGCACCACACCTTCTACAATGAGCTTCGTGTTGCTCCCGAAGAACACCCCGTCCTTCTCACCGAAGCTCCTCTCAATCC

> *BHLH*

CCAGCAGGTTGTCCTTGTGCAGCAAAGGCAGCTGCTGGTAATACAGGAGGGTGAGGCATGTAGCTCGGGCGAGCGCTCAACATCTTCACTTGTTGTTCCAGGTTCTCCTTCTCTTGCTTTAGCCTCTGCTTCTCATCACGGA

> *CLA*

GATGAGATTCTGATTGCTGGTGAGCTCCAGGAGTCGAGCAAGAAGACTGTGGCCAGACTCATAGCTGCTCAGGATTCCTTGGTGGAAACAGCCAAAGAGCAGG

> *CYP*

ACCCTTGGGCAAGAACAACAGAAGAATGGCAAGCTAACTGGAATGGCTTTCCGTGTTCCCACTGTCGATGTGTCTGTTGTTGATCTCACTGTTAGGCTTGAGAGCATCCTCCAACTCAACCTCCTTT

> *EF1*

GGCACTAACTCGCTCCTTCTGAGCTTCATCGGAGATATCCACTCTAGTCCACTCATACAGCTCCATGTCATAGCACTCATCAATCACAAACTGAGGGATCTCCTTCCCACGAAAAAGCCACAAGCCCTTGACCTTGAAGGGCGGCTCCGATCCAATCACCAGCATCTTACCAA

> *GAPDH*

CACGGTCAGTGGAAGCACCATGAGATAAAGGTGAAGGACTCGAAGACTCTCCTCTTTGGCGAGAAGCCAGTCACCATCTTTGGTGTTAGGAATCCTGAGGAGATCCCATGGGGTGAGGCTGGTGCTGACTTCGTTGTGGAGTCTACTGGTGTATTCACTGACAAGGATAAGGCTGCTGCT

> *SAND*

CCAATACCCAGATGAGGAGACAAAGATCCATATCCTCTCTTGTCCGGGCGTAGATAAGGAGATGGTGACCCAGAAGAATTAACTGGCAATTCCTCGACATGCAGACCACCATCTAACATGGATCTCTGAATTTCACACAGAACATTTGACTTCACAAGAACAGCCTCAATGCGAATCC

> *TIP41*

CGAAGCCAGAAACGGAGAAGAATAAACCAATAACTCGGCATCACTCTCACTTTTACAGTTAAGAGTGATACACCATTGTCAGCCAGCTCATCTTCGTACAAGACAACCTCGTCATAAAAGAGTATTGGTTCCTTCAATGATAGTGCAGCCATATCAATTTGCTCATTGCATTCTTCCCAATCCACCCTACCC
